# Autoantibodies in COVID-19: implications for disease severity and clinical outcomes

**DOI:** 10.3389/fimmu.2024.1509289

**Published:** 2025-01-06

**Authors:** Yannick Galipeau, Curtis Cooper, Marc-André Langlois

**Affiliations:** ^1^ Department of Biochemistry, Microbiology and Immunology, Faculty of Medicine, University of Ottawa, Ottawa, ON, Canada; ^2^ The Ottawa Hospital Research Institute, Ottawa, ON, Canada; ^3^ Centre for Infection, Immunity and Inflammation (CI3), University of Ottawa, Ottawa, ON, Canada

**Keywords:** SARS-CoV-2, COVID-19, autoantibodies, ACE2, autoimmunity, long-COVID, post-acute sequelae

## Abstract

Few pathogens have historically been subjected to as intense scientific and clinical scrutiny as SARS-CoV-2. The genetic, immunological, and environmental factors influencing disease severity and post-infection clinical outcomes, known as correlates of immunity, remain largely undefined. Clinical outcomes of SARS-CoV-2 infection vary widely, ranging from asymptomatic cases to those with life-threatening COVID-19 symptoms. While most infected individuals return to their former health and fitness within a few weeks, some develop debilitating chronic symptoms, referred to as long-COVID. Autoimmune responses have been proposed as one of the factors influencing long-COVID and the severity of SARS-CoV-2 infection. The association between viral infections and autoimmune pathologies is not new. Viruses such as Epstein-Barr virus and cytomegalovirus, among others, have been shown to induce the production of autoantibodies and the onset of autoimmune conditions. Given the extensive literature on SARS-CoV-2, here we review current evidence on SARS-CoV-2-induced autoimmune pathologies, with a focus on autoantibodies. We closely examine mechanisms driving autoantibody production, particularly their connection with disease severity and long-COVID.

## 1 Introduction

SARS-CoV-2 has profoundly impacted the lives of the global community since its emergence from Hubei province, China, in late 2019 (1, 2). This novel beta-coronavirus rapidly spread among humans and animals causing the COVID-19 pandemic. Fortunately, the prompt development of vaccines and extensive immunization efforts significantly reduced the mortality rate following infection. Nonetheless, over seven million deaths have so far been attributed to SARS-CoV-2 (3). Despite these medical advances, the incidence of breakthrough infections and vaccine hesitancy among some individuals means that SARS-CoV-2 continues to impose a significant burden on healthcare systems worldwide (4–7).

While most infected individuals return to their pre-infection health levels, some do not. These individuals continue to experience ongoing and/or new chronic symptoms for months after the initial infection (8–10). Although diagnostic guidelines remain unclear, these individuals are generally referred to as having post-acute sequelae of SARS-CoV-2, also known as long-COVID patients or long-haulers (11–13).

Another question that has stymied the scientific community is the highly heterogeneous nature of COVID-19 disease severity (14, 15). While many individuals experience mild or even asymptomatic infections (16, 17), others suffer severe infections requiring hospitalization, which can sometimes result in death (18, 19). Multiple studies have identified independent factors and co-morbidities that increase the likelihood of poor outcomes following infection, such as older age, respiratory illnesses, cardiovascular conditions, obesity, and weak antibody immunity (20, 21). However, these factors do not account for all severe clinical outcomes following infection. Given this conundrum, some have suggested that SARS-CoV-2-induced autoimmune pathologies may be a contributing factor (22–24). It has also been hypothesized that autoantibodies are a contributing factor to long-COVID (25).

The underlying factors governing the etiology and development of autoimmune responses and disease are not well understood and are believed to have roots in genetics, demographic characteristics (e.g., age and sex), and environmental factors (26–28). Viruses are considered one of several central exogenous factors able to trigger autoimmunity (26). Correlational studies have highlighted close links between certain viruses and the subsequent development of autoimmune pathologies. For example, Bjornevik et al. showed that individuals who seroconverted to Epstein-Barr virus (EBV) had a 32-fold increased risk of being diagnosed with multiple sclerosis (MS) (29). Other studies have also linked EBV infections to systemic lupus erythematosus (SLE), rheumatoid arthritis (RA), and other autoimmune pathologies (30–33). Type 1 diabetes mellitus is another example of a disease with possible linkages to viral infections and autoimmunity. Enteroviruses such as coxsackievirus and rotavirus have been linked with beta-cell autoimmunity (26, 34–36). Although multiple mechanisms have been proposed to explain viral-induced autoimmunity, the etiological link remains unclear due to the scarcity of underlying mechanistic evidence.

Here, we review the association between SARS-CoV-2 infection and autoimmune pathologies, emphasizing the production of various autoantibody classes. We present current experimental evidence on the presence and induction of these autoantibodies in the context of SARS-CoV-2 infection, and explore potential mechanisms underlying their emergence. Additionally, we address the complexities of establishing causal relationships between autoimmunity and disease processes, situating these findings within the broader context of SARS-CoV-2 disease severity and the evolving clinical picture of long-COVID.

## 2 Types of autoantibodies detected in SARS-CoV-2 infections

**Figure 1:**
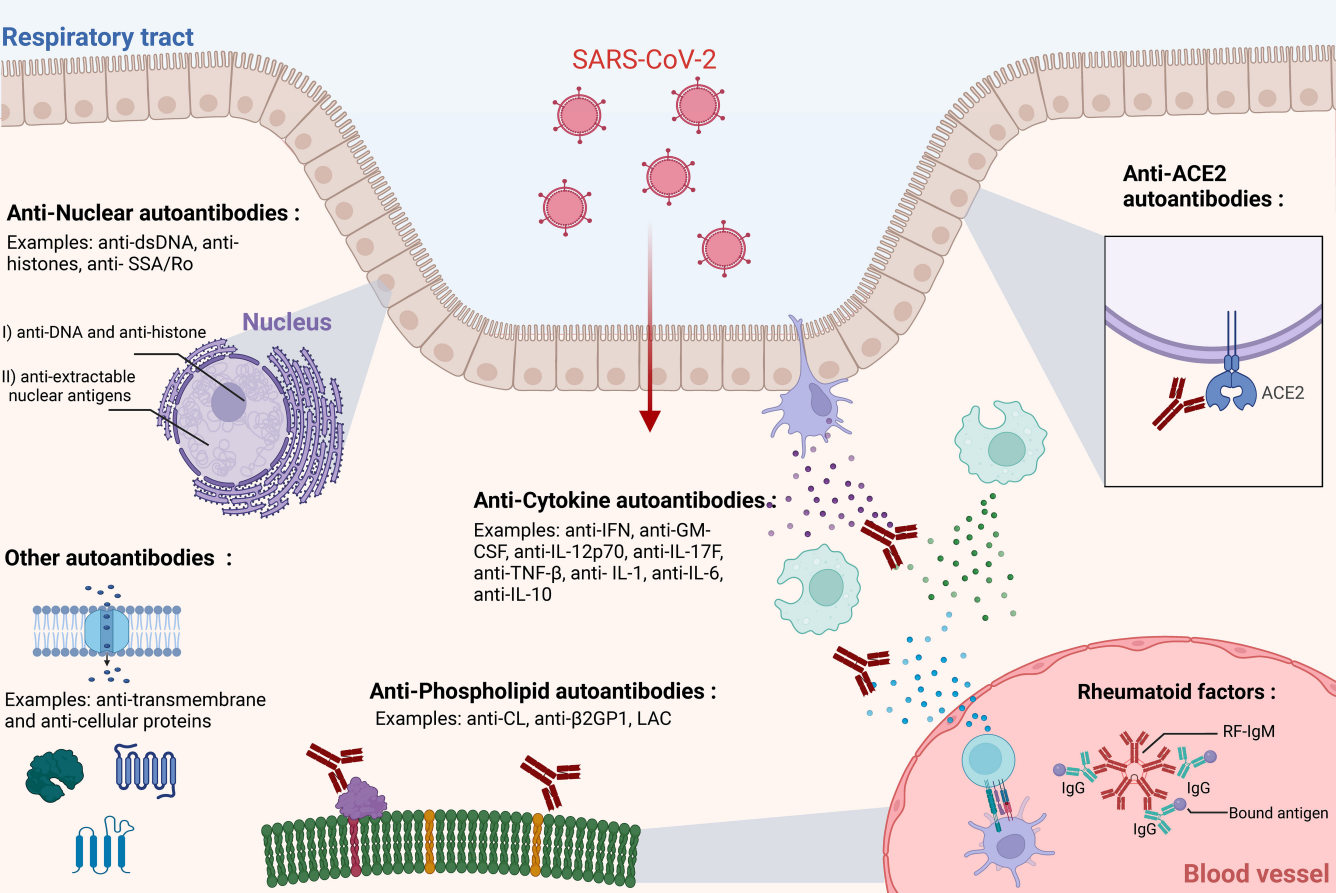
Types of autoantibodies associated with SARS-CoV-2 infection. Multiple studies have highlighted the association, induction, and possible roles of various types of autoantibodies in SARS-CoV-2 infection. This figure illustrates the primary categories of autoantibodies: Anti-ACE2, Anti-Cytokine Antibodies (ACAs), Antinuclear Antibodies (ANAs), and Anti-Phospholipid Antibodies (APLAs), Rheumatoid factors (RFs). Autoantibodies can generally be detected in circulation but may act in specific physiological locations (e.g., blood, respiratory tract epithelium). For simplicity, IgG antibodies are mostly depicted here, although IgM and IgA antibodies may also be present. The respiratory epithelium layer is shown as a representative site for SARS-CoV-2 infection, although the virus can infect multiple other tissues. The figure was created in https://BioRender.com.

Autoantibodies are immunoglobulins that recognize and bind to self-antigens (e.g., serum proteins, cellular proteins, phospholipids, nucleic acids) (37–39). These antibodies, which seem to defy tolerance mechanisms, have been detected in many vertebrates including sharks, fish, turtles, mice, and humans (40–43). Their ubiquitous prevalence suggests a broader role in homeostasis (44). Some propose that autoantibodies are important for immune regulation and function, clearance of apoptotic cells, transport and modulation of biologically active molecules, and other physiological processes (45). Autoantibodies can differ in isotype and binding properties. Some can bind multiple self-antigens with varying affinities and can be constituted of minimally mutated recombined V(D)J gene sequences, while others have undergone more intense somatic hypermutation resulting in higher affinity autoantibodies (46). While these antibodies can occur in healthy individuals without any obvious impact on health, environmental exposures such as viral infections can sometimes trigger the generation of pathogenic autoantibodies or increase existing autoantibodies to pathogenic levels. This section summarizes experimental evidence and describes various potentially pathogenic autoantibodies linked with SARS-CoV-2 infections (**Figure 1**).

### 2.1 Anti-nuclear antibodies

Antinuclear antibodies (ANA) are a well-known group of autoantibodies that recognize and bind to antigens within the nuclear compartment (47–49). These autoantibodies are generally classified into two large groups: (I) antibodies targeting DNA and histones, and (II) antibodies targeting extractable nuclear antigens, including RNA-binding proteins complexed to RNA (50, 51). Examples of ANA antibodies include anti-dsDNA, anti-histones, anti-Smith antigen, anti-SSA/Ro, anti-SSB/La, anti-Scl70, and anti-Jo-1 (51). Their combinatorial presence has been used as laboratory biomarkers for several autoimmune diseases such as rheumatoid arthritis (RA), systemic lupus erythematosus (SLE), Sjögren’s disease, and systemic sclerosis (50, 52–54).

The prevalence of ANA autoantibodies in SARS-CoV-2 infections has been explored by multiple groups. Early in 2020, studies reported ANA seroprevalence in small cohorts of SARS-CoV-2-infected individuals that ranged from 34.5% to 54.5% (55–57). Larger cohorts estimated ANA prevalence at around 35.6% by Gazzaruso et al., around 25% by Lerma et al., and around 57.5% by Sacchi et al. (58–60). Interestingly, ANA autoantibodies cannot simply be explained by hyperglobulinemia, as they are produced in different proportions to total immunoglobulin levels in plasma (61). The notable heterogeneity in seroprevalence of ANA antibodies across studies could be explained by SARS-CoV-2 severity and cohort characteristics. For example, Taeschler et al. observed a higher trend of ANA antibodies in individuals with severe COVID-19 compared to those with milder infections (62). Despite numerous seroprevalence studies on ANA, mechanistic studies are limited. Recently, a longitudinal cohort study of 106 convalescent individuals identified ANA antibodies as a predictor of long-COVID (63). Interestingly, in another large recent study, the authors found an increased risk of an autoimmune pathology diagnosis after COVID-19 in those with detectable ANA antibodies (64). Nonetheless, it is important to note that ANA antibodies are also found in 14-27% of healthy individuals without symptomatic presentations (65–68). The low predictive value of positive antinuclear antibodies (ANA) requires careful correlation with clinical findings to support an autoimmune diagnosis. Consequently, studies linking SARS-CoV-2 infection with ANA positivity must be interpreted within the context of the study’s healthy control cohorts, the specific assays used, and the established positivity thresholds.

### 2.2 Anti-cytokine antibodies

Antibodies recognizing various cytokines have been described in the context of viral infections prior to the COVID-19 pandemic (69, 70). Anti-cytokine antibodies (ACA) have been shown to bind a vast array of common cytokines such as IL-1, IL-6, IL-8, IL-12, GM-CSF, IFN-α, IFN-γ, and TNF-α (71–80). Interestingly, certain categories of anti-cytokine antibodies have been linked with disease. For example, anti-GM-CSF autoantibodies are believed to be responsible for idiopathic pulmonary alveolar proteinosis (PAP). While primary PAP is caused by a mutation in the GM-CSF receptor, idiopathic PAP is caused by the neutralization of GM-CSF itself (81–83). Anti-cytokine autoantibodies, particularly anti-IFN-γ, have been linked with poor clinical outcomes and increased disease severity in individuals infected with nontuberculous mycobacteria and other opportunistic infections (84–87). These antibodies can effectively neutralize interferon-gamma, leading to poor immune engagement and an overall compromised immune response (84, 88). Immune suppression by ACA has led to their inclusion in the IUIS classification of primary immunodeficiency diseases, commonly referred to as adult-onset immunodeficiency syndrome (AOID) (89, 90). Bastard et al. first reported that in a cohort of 987 individuals with severe COVID-19, 10.2% had detectable levels of autoantibodies against type I IFN. These autoantibodies were not found in individuals with mild or asymptomatic SARS-CoV-2 infections. The overall seroprevalence of these autoantibodies in healthy individuals was approximately 0.33%, underscoring a significant association with severe COVID-19 (91). These observations have been confirmed by other groups across various demographics (92–96). Anti-IFN antibodies are thought to pre-exist in certain individuals rather than being induced by infection. This highlights their potential to exacerbate common infection severity through IFN neutralization, thereby inhibiting downstream antiviral signaling pathways (91). Other types of ACA have also been identified in severe cases of SARS-CoV-2. For example, Chang et al. used a multiplex protein array to identify antibodies targeting IL-1, -6, -10, -15, -17A, -22, -21, MIP-α, and VEGF-B (61). Another group reported similar findings, including IFN-γ, GM-CSF, IL-12p70, IL-17F, and TNF-β, but only found anti-IL-12p70, anti-IL-22, and anti-IL-6 to be neutralizing. Non-neutralizing anti-cytokine antibodies can still have clinical relevance; however, their role in SARS-CoV-2 infection is not well defined. Key questions remain regarding the triggers for their induction and the persistence of ACA levels over time. Additionally, the clinical impact of most ACAs is poorly understood. In the context of long-COVID, there is currently no evidence to suggest that ACAs contribute to the condition.

### 2.3 Anti-ACE2 antibodies

Intensive research has centered on the angiotensin-converting enzyme 2 (ACE2) protein, recognized as the entry receptor for both SARS-CoV-1 and SARS-CoV-2. Additionally, ACE2 serves as the entry receptor for NL63, a common human alphacoronavirus (97). Given the key role of this protein in viral entry, some hypothesized that auto-reactivity to ACE2 could contribute to severe COVID-19 symptoms (22, 98). Building on these questions, several studies examined anti-ACE2 autoantibodies in SARS-CoV-2-infected individuals. Arthur et al. found that antibodies recognizing ACE2 were detected in 93% of inpatients, and 81% of convalescent controls, but in none of the healthy controls (99). Another study found IgM antibodies to ACE2 in 27.2% of individuals with severe infections but only 3.8% of those with mild disease, similar to the control cohort (4%) (100). Additional studies have documented the presence of anti-ACE2 antibodies (101, 102). However, anti-ACE2 antibodies have not shown significant capacity to inhibit ACE2 enzymatic activity (99, 100). Interestingly, a study demonstrated the induction of anti-ACE2 antibodies following immunization with recombinant RBD in mice (101). While there is evidence that SARS-CoV-2 infection can induce anti-ACE2 autoantibodies, the topic remains controversial. For example, Chang et al. did not find elevated anti-ACE2 autoantibodies in hospitalized SARS-CoV-2 patients compared to other autoantibodies (61). In a cohort study of 464 individuals, anti-ACE2 IgG autoantibodies were detected in 10.3% of participants, IgA in 6.3%, and IgM in 18.8%, with no association found with SARS-CoV-2 vaccination or infection (103). ACE2 autoantibodies are not exclusive to SARS-CoV-2 infection, having been detected in conditions such as Parkinson’s disease, vasculopathy, and rheumatoid arthritis (104–108). The presence and function of anti-ACE2 antibodies in serum remain unclear, with mixed evidence of their association with SARS-CoV-2 infections.

### 2.4 Anti-phospholipid antibodies

Anti-phospholipid antibodies (APLAs) target phospholipids and phospholipid-binding proteins such as cardiolipin (CL), β2-glycoprotein-1 (β2GP1), and non-classical antigens like prothrombin (PT) and annexin-V (109–113). These autoantibodies are primarily associated with antiphospholipid syndrome (APS), a condition characterized by thrombotic events and pregnancy complications (113, 114). While murine models have demonstrated APLA pathogenicity in APS, the underlying etiology remains unclear (115, 116).

Historically, APLAs were first identified in fetuses with congenital syphilis in 1906 and have since been linked to infections such as HIV and HCV (117, 118). COVID-19 coagulopathy shares similarities with thrombotic events observed in both standard and catastrophic APS (119, 120). In early 2020, a case report suggested a potential role for APLAs in severe COVID-19 (121). Subsequent studies have further explored this association. For example, critically ill COVID-19 patients were reported to exhibit elevated levels of APLAs, such as IgA against CL and IgG/IgA against β2GP1 (122).

While several other studies have also highlighted the prevalence of elevated APLAs in COVID-19 patients (59, 123–125), Trahtemberg et al., however, found that APLAs associated with increased disease severity of patients with respiratory failure regardless if they were infected by SARS-CoV-2 or not (126). Reports have also correlated APLAs with thrombosis. Helms et al., for instance, identified APLAs in 87.7% of 57 SARS-CoV-2 patients with coagulation abnormalities (127). Supporting evidence from other studies has confirmed similar findings (122, 128).

Mechanistically, Zuo et al. demonstrated that IgG fractions from COVID-19 patients with APLAs induced neutrophil extracellular trap (NET) release in healthy neutrophils and promoted venous thrombosis in mice (129). However, Serrano et al. observed elevated anti-β2GP1 levels in COVID-19 patients but found no association with thrombosis (130). Additionally, some studies reported no link between APLAs and thrombosis (123, 124, 131).The relationship between COVID-19-associated coagulopathy and APLAs remains ambiguous. It is unclear whether these coagulopathies are independent of APLAs, whether APLA prevalence is specific to COVID-19, or if it is simply a feature of severe illness and hospitalization. Uncharacterized APLAs may play a role in these coagulopathies. Notably, baseline APLA levels vary with age and may be influenced by other infections or autoimmune disorders (118, 132, 133).

### 2.5 Rheumatoid factors

Rheumatoid factors (RFs) are a common class of autoantibodies that recognize and bind the Fc region of IgG immunoglobulins (**Figure 1**) (134, 135). RFs can be of any isotype and have been described extensively in the context of rheumatoid arthritis but have also been associated with other autoimmune conditions (e.g., SLE, Sjögren’s disease) (136–138). Notably, RFs have also been described in the context of viral infections, such as hepatitis C virus (HCV) (139, 140). Recently, RFs have been linked to COVID-19. In a cohort of 129 individuals, Xu et al. detected RFs in 20% of individuals infected with COVID-19 (141). Other studies have reported a variable RF seroprevalence among COVID-19 patients, ranging from 20% to 50% (141–144). One study found higher rates of death, ICU admission, and mechanical ventilation in COVID-19 patients that were RF-positive, suggesting a link with disease severity (142). Interestingly, another study identified novel polyreactive RFs in COVID-19, with one RF able to bind epitopes on IgG and the spike protein (145). The role of RFs in SARS-CoV-2 infection and recovery remains unclear. Previous research has suggested that RFs may neutralize virus-antibody complexes (146) and enhance viral clearance (147). Additionally, RF-positive B cells have been shown to present antigens to T cells, potentially augmenting cellular immune responses during viral infections (148).These findings raise the possibility that RFs may play a protective role during SARS-CoV-2 infection; however, further mechanistic studies are required to elucidate their exact function.

### 2.6 Other types of autoantibodies

Anti-citrullinated protein antibodies (ACPAs) are a notable class of autoantibodies that recognize peptides containing citrulline, a post-translational modification of arginine (149). ACPAs are strongly associated with autoimmunity and serve as a hallmark marker of rheumatoid arthritis (150–152). Early in the COVID-19 pandemic, a case study reported a potential association between ACPAs and SARS-CoV-2 infection (153). However, subsequent studies have produced mixed results, with some supporting a connection between ACPAs and COVID-19, while others have not found a significant link (154–156).

Another group of autoantibodies relevant to SARS-CoV-2 infections are anti-neutrophil cytoplasmic antibodies (ANCAs). These autoantibodies are primarily associated with ANCA-associated vasculitis (AAV) (157). Case reports have documented instances of AAV occurring in the context of SARS-CoV-2 infection (158, 159). However, the association of both ACPAs and ANCAs with COVID-19 requires validation through studies involving larger cohorts.

Recent advancements in high-throughput multiplexed approaches have significantly enhanced our understanding of autoantibody prevalence. For instance, human exoproteome display analysis using a yeast library has facilitated the profiling of several hundred potential autoantigens. These techniques have been applied to studies of SARS-CoV-2 infection and long-COVID. Interestingly, Klein et al. utilized these methods to evaluate autoantibodies against thousands of putative self-antigens. They observed no significant differences in autoantibody levels or specific enrichment in individuals with long COVID (160). However, in severe COVID-19 cases, the same approach revealed a broad range of autoantibodies targeting lymphocyte function, cytokines, complement factors, growth factors, cell surface proteins, and more (95). Other high-throughput approaches confirmed that severe SARS-CoV-2 infections induce autoantibodies against a broad array of secreted and non-secreted proteins (61, 161, 162). These unbiased assays provide a comprehensive assessment of circulating autoantibody distributions.

## 3 Mechanisms for autoantibody induction during viral infection

**Figure 2:**
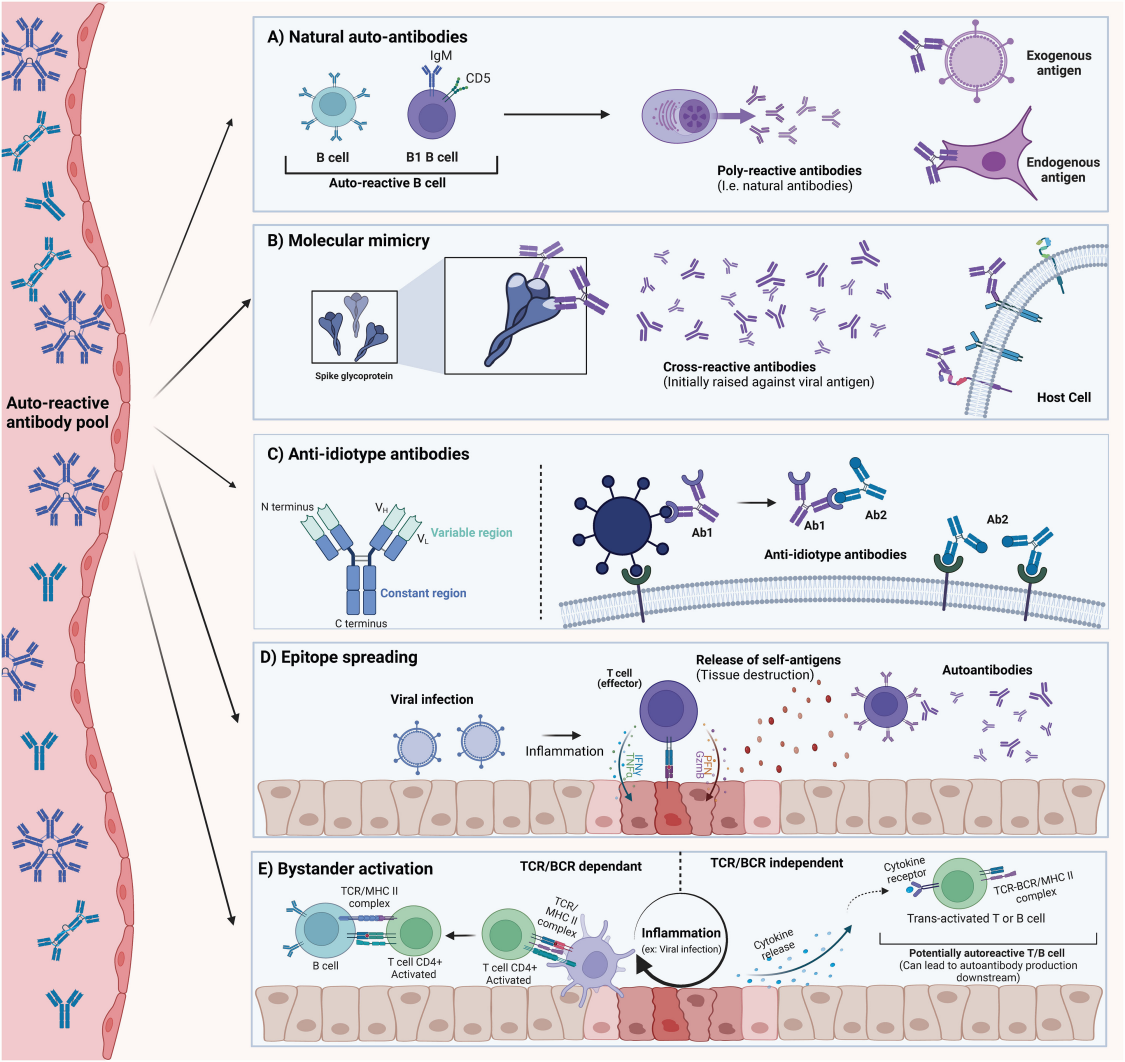
Description of possible mechanisms for autoantibody generation. These mechanisms should be considered as non-exclusive, as more than one mechanism can explain the pool of autoantibodies detected in SARS-CoV-2. **(A)** Natural Autoantibodies can be found in most vertebrates and seem to be important in normal physiological processes. They are believed to arise mainly from the B1 cell subset. These autoantibodies usually exhibit poly-reactivity to endogenous and exogenous proteins. **(B)** Molecular Mimicry occurs when an epitope is shared between an exogenous antigen and an endogenous protein. This can result in cross-reactive autoantibodies that bind to host-derived proteins. **(C)** Anti-Idiotype Autoantibodies (Ab2) are generated against the variable region of another antibody (Ab1). This antibody (Ab2) can potentially retain structural determinants of the original antigen, and in some cases, could bind its target. **(D)** Epitope Spreading (Intermolecular) can occur when an ongoing inflammatory process (initiated by a viral infection) results in the release of normally sequestered self-antigens. T and B cell specificity can then extend to self-antigens that would not normally be presented. **(E)** Bystander Activation refers to the loss of TCR/BCR engagement requirement for activation that can happen to immune cells in proximity to a highly inflammatory milieu. Other signals (such as cytokine release) decouple the need for proper engagement with innate cells and BCR/TCR-MHC interaction. This can result in the activation of auto-reactive clones. The figure was created in https://BioRender.com.

Studies on the prevalence of autoantibodies across various health conditions, including viral infections such as SARS-CoV-2, are relatively common; however, the mechanisms leading to increased self-tolerance breakdown during viral infections remain unclear. In this review, we describe, explain, and discuss the most relevant mechanisms thought to drive autoantibody production and potential pathogenesis in the context of viral infections (**Figure 2**). These mechanisms are not mutually exclusive, and multiple pathways may contribute to the pool of circulating autoantibodies. While T cells are also crucial in the generation and maintenance of autoantibodies, their role is beyond the scope of this review.

### 3.1 Natural autoantibodies

Natural autoantibodies are commonly occurring, low-specificity, and polyreactive antibodies found in all vertebrates (44, 163–165). These antibodies can occur independently of antigenic encounters and are able to recognize both endogenous and exogenous antigens (43, 166, 167). Networks of natural antibodies have been proposed to be remnants of evolution prior to the appearance of specific adaptive immune responses (163, 168). The presence of natural autoantibodies raises critical questions about our understanding of self-tolerance mechanisms. Central and peripheral tolerance mechanisms during B cell development are designed to eliminate autoreactive clones. For instance, in the bone marrow, negative selection of immature B cells upon recognition of self-antigens can lead to B cell receptor (BCR) editing (169). In addition, auto-reactive immature B cells that are unable to rescue a self-reactive BCR may undergo apoptosis by a mechanism known as clonal deletion (170). Despite this, a large number of self-reactive clones persist in the periphery where other tolerance checkpoints are in place such as B cell anergy (171). Nevertheless, the immune system appears capable of maintaining a repertoire of B cell receptors that are polyreactive and can secrete polyreactive immunoglobulins. These are believed to be secreted primarily by B1-cells co-expressing CD5, although other subsets have been reported (172–175). Given this, it is crucial to consider that some of the autoantibodies detected during and after SARS-CoV-2 infection may be natural autoantibodies or could result from the expansion or upregulation of polyreactive B cells already present in circulation due to inflammation or immune system activation during infection. A better characterization of the affinities and rate of somatic hypermutation, as well as identifying B-cell subsets able to generate such autoantibodies is critical to understand if natural antibodies play a key role in SARS-CoV-2 related autoantibodies.

### 3.2 Molecular mimicry

Molecular mimicry is one of the most widely recognized mechanisms associated with autoimmunity. In its simplest form, it refers to the phenomenon where certain exogenous antigens, such as viral proteins, including those from SARS-CoV-2, share common epitopes with endogenous antigens (26, 176–178). Antibodies raised against these regions of high homology can in some cases lead to cross-reactive antibodies that can be pathogenic to the host (179–181). In 1962, the work and observations of Kaplan et al. showed that antibodies against group A streptococci can cross-react with cardiac tissues, leading to the death of an individual due to rheumatic pancarditis caused by a streptococcal infection (182). In the following years, multiple lines of evidence led to the acceptance of molecular mimicry as a mechanism explaining cross-reactive antibodies following infection. In fact, molecular mimicry has been observed with various viruses such as Influenza, Zika, Epstein-Barr virus (EBV), and others (183–189). For instance, mimicry between EBV nuclear antigen 1 (EBNA1) and the central nervous system protein glial cell adhesion molecule (GlialCAM) has been shown to generate cross-reactive antibodies, which are detected in approximately 25% of multiple sclerosis (MS) patients (190).

Recent computational analyses of structural homology between SARS-CoV-2 antigens and the human proteome have identified several shared epitopes. For example, Kanduc et al. identified several linear hexapeptide epitopes that displayed conserved homology between SARS-CoV-2 proteins and 460 human proteins (191). Other groups, using slightly different approaches, have also reported several SARS-CoV-2 proteins containing regions that can be referred to as “molecular mimicry hot-spots” (192–194). Interestingly, immunization of mice with the receptor-binding domain (RBD) protein, as demonstrated by Lai et al. generated antibodies capable of binding to ACE2, suggesting the presence of cross-reactive epitopes (101). Validation of these findings from animal models in larger cohorts of vaccinated individuals remains to be conducted. Importantly, molecular mimicry is not a binary “all-or-nothing” model; genetic factors, such as HLA haplotypes, and environmental factors, such as infections, can influence antigen processing and peptide presentation (195).

### 3.3 Anti-idiotype antibodies

Anti-idiotype antibodies are not often discussed in the context of autoimmunity. However, these types of antibodies remain interesting as they could explain, at least partially, some of the autoantibodies detected during SARS-CoV-2 infections. The concept of anti-idiotype antibodies, first introduced by Niels Jerne in 1974, suggests that antibodies generated in response to infection (Ab1) possess an immunogenic component within their variable regions that constitutes the idiotype. This region could then be recognized as an antigen, and as such, new antibodies (Ab2) generated against this region would structurally mimic the original antigen (196). This can hypothetically have ramifications whereby anti-idiotype Ab2 antibodies could bind membrane-bound proteins of the host, form immune complexes, and potentially drive pathogenic effects (197, 198). In the context of the SARS-CoV-2 spike protein, one can hypothesize that antibodies against the spike protein could represent the Ab1, and newly generated Ab2 antibodies would be able to bind Ab1 antibodies, but also potentially the ACE2 protein. Interestingly, anti-idiotype antibodies have been explored as possible vaccine candidates for infectious diseases and cancer therapies (199–203). Despite this intriguing hypothesis, at the time of writing this review, no experimental evidence has been reported to support this mechanism in the context of SARS-CoV-2.

### 3.4 Epitope spreading

Epitope spreading (ES) can be described as the broadening of reactive lymphocytes to other antigen/epitopes (204, 205). Epitope spreading can be subdivided into intramolecular ES when reactive lymphocytes are able to react with cryptic, non-presented, non-available, or sub-dominant epitopes (206, 207). In contrast, ES can also occur through the diversification of reactive T and B lymphocytes toward antigens distinct from the initially presented antigen that triggered their expansion, a phenomenon commonly referred to as intermolecular ES. Intermolecular ES is frequently discussed in contexts of tissue damage, whether caused by direct trauma or by tissue destruction through phagocytic and inflammatory mechanisms (208, 209). Several factors influence the magnitude of ES, some of which include the type and intensity of the primary inflammatory process and the magnitude of the tissue damage (210). It is also important to consider that ES can be a normal feature of immunity, allowing for more efficient and diverse adaptive responses (208). While ES has been associated with multiple viruses, whether ES contributes to SARS-CoV-2-related autoantibodies remains inconclusive (26, 211). However, tissue damage has been extensively described in severe COVID-19 (212, 213). As such, it is reasonable to suggest that ES is a valid possibility that could explain the generation of some of the autoantibodies identified following SARS-CoV-2 infections.

### 3.5 Bystander activation

Bystander activation has been proposed as a mechanism that may explain the activation of auto-reactive lymphocytes independently of their BCR/TCR specificity (214, 215). This antigen-independent activation relies on several co-stimulatory signals that decouple the requirement of BCR/TCR signaling with their specific antigen (214, 216). Some of these signaling mediators include ligands (co-signaling receptors, pathogen-associated molecular patterns), cytokines, and chemokines (214, 217–221). These signaling mediators can all occur during infection, resulting in a localized pro-inflammatory environment that can trigger bystander activation of nearby lymphocytes. For example, during primary HIV infection, CD8+ T cells against influenza, EBV, and CMV show markers of activation and expansion in some individuals, despite the absence of cognate antigens (219). Several autoimmune disorders have been associated with bystander activation such as rheumatoid arthritis (RA), systemic lupus erythematosus (SLE), Grave’s disease, and Hashimoto’s thyroiditis (222–225). While direct evidence of bystander activation in SARS-CoV-2 infections is limited, it is well known that severe infections have been correlated with an overall release of pro-inflammatory cytokines (226, 227), which have the potential to initiate bystander activation in some individuals.

### 3.6 Other proposed mechanisms

In addition to the mechanisms discussed in this review, several other processes may contribute to the generation of autoantibodies. These include direct infection of lymphocytes, the activity of superantigens, and the breakdown of immune tolerance mechanisms (228, 229). It is also important to recognize that polyreactive antibody-secreting cells can positively contribute to antibody-mediated immune responses during infection. For instance, in the context of influenza infections, polyreactive monoclonal antibodies (mAbs) have demonstrated increased binding breadth to antigenically drifted and shifted influenza A virus (IAV) antigens (230). Similarly, in HIV infections, two well-characterized broadly neutralizing mAbs were found to be polyreactive and cross-reactive to cardiolipin (231).

## 4 Possible regulatory roles of autoantibodies

Autoantibodies have also been linked to the relief of inflammatory pathologies, suggesting possible regulatory functions. It is important to consider that autoantibodies may not all be pathogenic, and some may possibly be important features of normal physiology. For example, Sjöwall et al. showed that in patients with systemic lupus erythematosus (SLE) a reduction of TNFα autoantibodies was linked with disease exacerbation (78). Similarly, another group showed that autoantibodies against type 1 IFN in SLE correlated with lower levels of IFN bioactivity and reduced downstream IFN pathways, which correlated with a lower disease score (77). It is also important to consider the regulatory role of antibodies in the treatment of numerous autoimmune or inflammatory conditions. For example, Guillain-Barré syndrome, chronic inflammatory demyelinating polyneuropathy, vasculitis, immune thrombocytopenic purpura, and several others are often treated with intravenous immunoglobulins (IVIG) (232–234). Anti-idiotype interactions, inhibition of complement deposition, saturation of the neonatal Fc receptor (FcRn) involved in antibody recycling, and competitive blockade of activating Fc gamma receptors are all non-exclusive mechanisms proposed to explain the effects of intravenous immunoglobulin (IVIG) (233, 235). These examples highlight that antibodies, including autoantibodies, can be important in controlling aberrant or excessive inflammatory processes. Whether autoantibodies associated with SARS-CoV-2 are of regulatory importance in viral infections or post-infection physiological processes remains unclear but represents an interesting proposition.

## 5 Link with disease severity

Research on autoantibodies in individuals infected with or recovering from SARS-CoV-2 has primarily focused on those with severe COVID-19, aiming to elucidate their role in adverse outcomes. As a result, most studies have reported autoantibody presence in hospitalized patients with severe disease, leaving limited data on those with mild or asymptomatic infections. The definitive relationship between autoantibody production and disease severity remains under investigation.

A recent study stratified participants by disease severity, including healthy controls, and found an increased prevalence of autoantibodies—particularly those targeting cardiolipin, claudin, and platelet glycoproteins—with escalating disease severity and advancing age (236). Mechanistically, severe SARS-CoV-2 infections may result in a temporary loss of T cell tolerance. Woodruff et al. demonstrated that severe COVID-19 is associated with the expansion of antibody-secreting B cell populations with low somatic hypermutation, which contract upon recovery, indicating a transient period of reduced selection pressure (229). Similar findings have been observed in acute respiratory distress syndrome caused by bacterial pneumonia, suggesting this phenomenon may represent a physiological response to severe pulmonary infections (229). Recent data from Jaycox et al. suggest that the increase in autoantibodies seen in SARS-CoV-2 infections is not a feature of mRNA vaccinations, suggesting that severe infection may well be the cause (237). While anecdotal reports and small cohort studies have identified autoantibodies after vaccination (238, 239), vaccination remains protective against the development of autoimmune diseases (240, 241).

The underlying mechanisms driving autoantibody production during SARS-CoV-2 infection remain unclear. It is hypothesized that factors such as the intensity of the immune response, inflammation (e.g., cytokine storm), or viral proteins may contribute to their development. Variability in symptom profiles and infection characteristics, such as viral variants, may also influence autoantibody production.

Interestingly, one study highlighted that autoantibodies are not unique to SARS-CoV-2 but are a common feature in critically ill patients with non-SARS-CoV-2 respiratory infections (242).Anti-cytokine autoantibodies were more prevalent in critically ill patients with non-SARS-CoV-2 infections compared to non-infected critically ill individuals (242). Although baseline levels of anti-cytokine autoantibodies (ACAs) in SARS-CoV-2 patients remain understudied, ACAs are recognized as potential risk factors for severe disease. Future longitudinal studies are needed to quantify the risk of severe COVID-19 associated with pre-existing ACAs and to monitor whether specific ACAs are induced during infection.

## 6 Interpretation and considerations related to SARS-CoV-2-induced autoantibodies

**Figure 3:**
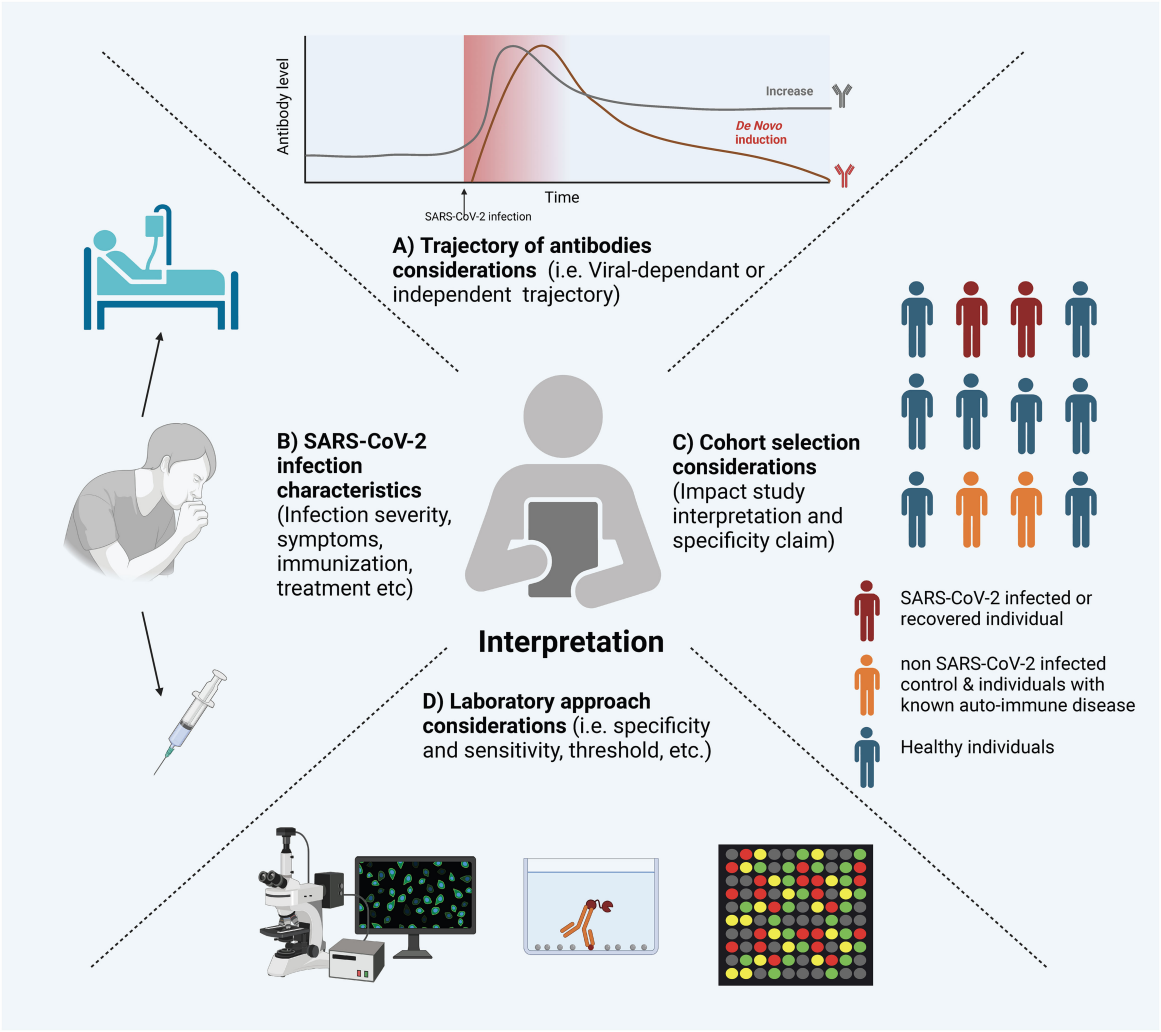
Considerations for Interpreting Experimental Evidence of Autoantibodies Following SARS-CoV-2 Infection. Several factors can influence the interpretation of SARS-CoV-2 induced autoantibodies. Here is a visual representation of a non-exclusive list of factors: **(A)** The Trajectory of Autoantibodies: Understanding whether autoantibodies are present chronically, induced independently of SARS-CoV-2, or induced by SARS-CoV-2 infection. **(B)** SARS-CoV-2 Infection Characteristics: Factors such as infection severity, treatment, and immunization status can significantly influence the presence and levels of autoantibodies. **(C)** Cohort Selection: The selection of cohorts, including characteristics like age, sex, underlying health conditions, and control groups, can have a substantial impact on the interpretation of results. **(D)** Laboratory Considerations: The experimental methods employed can significantly influence the sensitivity and specificity of autoantibody detection. The figure was created in https://BioRender.com.

The COVID-19 pandemic has highlighted the established link between viral infections and autoantibodies with a remarkable volume of literature emerging on this topic. Reaching robust conclusions about the causative factors behind autoantibody production and their physiological roles is challenging due to the wide diversity of study designs (e.g., cross-sectional, longitudinal, retrospective, case reports), laboratory assay methodologies, and the varying characteristics of SARS-CoV-2 infections and clinical outcomes (e.g., disease severity, hospitalization, viral variants, therapeutic interventions, vaccination status, and co-morbidities) (**Figure 3**).

### 6.1 Interpretation of laboratory assay data

The wide range of validated and in-house assays used to measure autoantibodies results in variability in reporting and interpretation, which often depends on the specific method employed. Taking ANA detection as an example, it has traditionally been performed using indirect immunofluorescence (IIF), which is considered the gold standard method (243). However, the development of alternative methods, such as solid-phase assays and bead-based multiplex platforms, has introduced challenges in standardizing results across different techniques (244). Beyond the challenges of standardization, each experimental system has distinct sensitivity and specificity profiles. While most assays used in clinical settings have been validated, in-house assays are inherently more flexible but their performance more heterogeneous. Therefore, it is essential to carefully select the assay type based on the specific research questions being asked. Furthermore, the choice of thresholds for seroprevalence measurements should be considered when comparing findings across different studies.

### 6.2 Pathogenicity potential of autoantibodies

**Figure 4:**
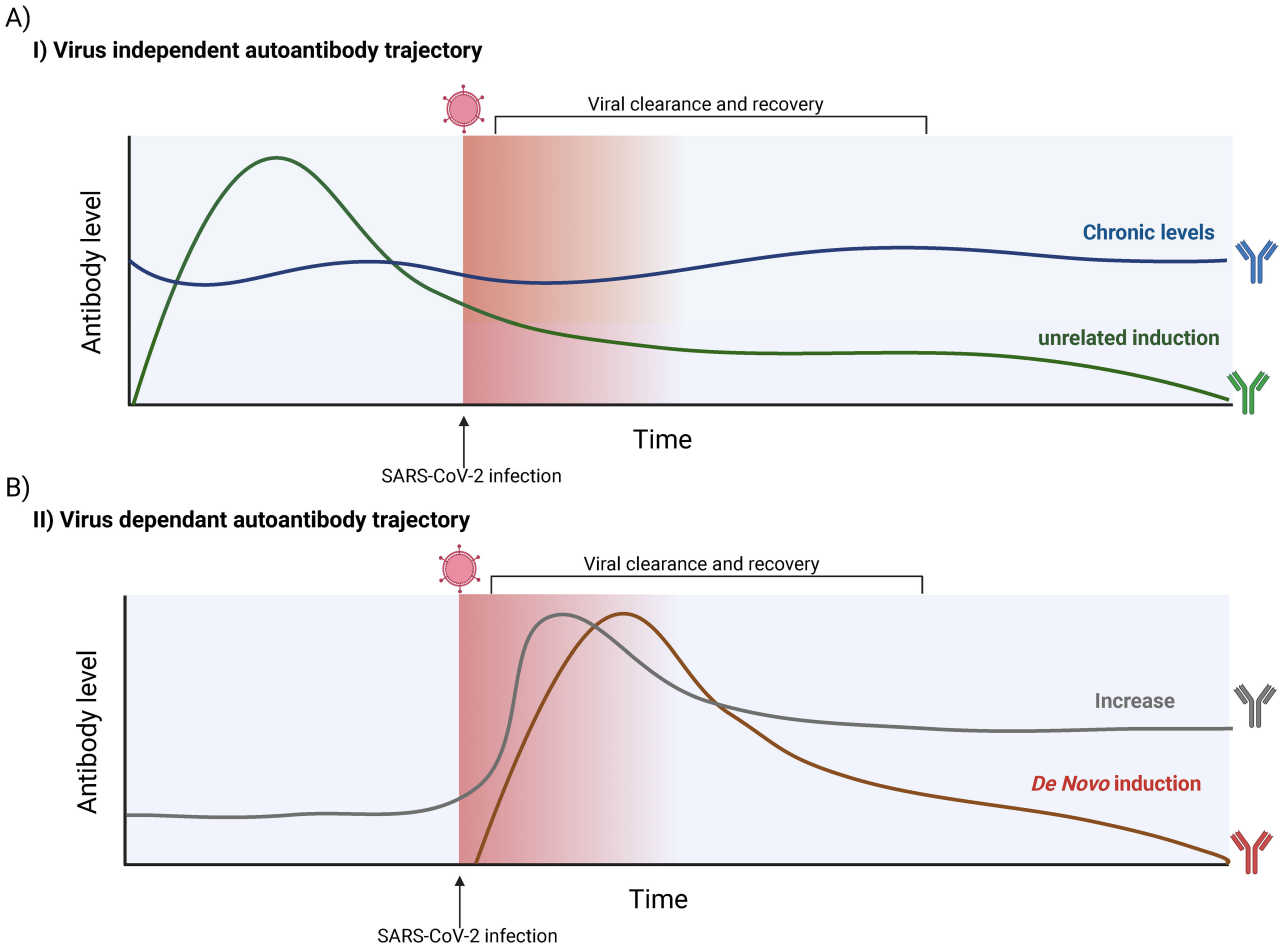
Possible SARS-CoV-2-dependant and -independent trajectories of autoantibody production. **(A)** Hypothetical trajectories of virus-independent autoantibodies. Autoantibody levels are not influenced by SARS-CoV-2 infection. Such antibodies are generally already present but can vary in level. A chronically present autoantibody (Blue) can remain relatively stable over time, with slight biological fluctuations. A SARS-CoV-2-independent induction can also occur (Green), in which antibody levels can rapidly increase and possibly decay over time. **(B)** Hypothetical trajectories virus-dependent autoantibodies detected in individuals. The presence and/or quantity of an autoantibody is influenced by SARS-CoV-2 infection. It is possible to see an increase in the titer of an autoantibody already detected in the infected host (Grey). This trajectory contrasts with a *de novo* induction, where SARS-CoV-2 infection leads to the induction of an autoantibody that was not in circulation prior to the infection (Red). These features of possible trajectories need to be considered when interpreting experimental evidence. The figure was created in https://BioRender.com.

The presence of autoreactive antibodies during and after SARS-CoV-2 infection is of significant clinical interest, but whether these antibodies are directly induced by the virus remains unclear. For example, Lebedin et al. demonstrated that some autoantibodies associated with COVID-19 are non-specific, polyreactive IgG. These polyreactive antibodies can interfere with the accurate quantification of target-specific and functionally relevant autoantibodies, which may have greater clinical importance (245). Additionally, the profiles of these autoantibodies before, during, and after infection are becoming increasingly well-characterized, enabling the proposal of distinct autoantibody trajectories (**Figure 4**).

Observational studies and those with limited control cohorts often cannot establish whether SARS-CoV-2 infection directly triggers autoantibody production. Given the heterogeneity of autoantibodies observed across various diseases, it is plausible that some findings may be unrelated to SARS-CoV-2 infection (246). For cases where a link between SARS-CoV-2 and autoantibodies has been demonstrated, a key question remains: are these autoantibodies induced *de novo*, or are pre-existing antibodies elevated to higher titers? For instance, Bastard et al. showed that autoantibodies targeting interferons (IFNs), associated with severe COVID-19, were present prior to infection but did not cause symptoms (91). This indicates that such autoantibodies may preexist in predisposed individuals. Further longitudinal studies that assess baseline autoantibody levels are needed to clarify their trajectories over time and elucidate their origins.

It is important to understand that the presence of autoreactive antibodies does not automatically indicate a causal relationship with the onset of autoimmune diseases. Notably, conditions associated with autoimmunity, such as myocarditis, arthritis, vasculitis, and encephalitis, have been reported following SARS-CoV-2 infection (247, 248). Retrospective studies in unvaccinated individuals have confirmed that SARS-CoV-2 infection significantly increases the risk of developing autoimmune disorders (240, 247). One study reported a 42.6% higher likelihood of autoimmune disease in individuals previously infected with SARS-CoV-2 (249). However, no definitive mechanistic studies have linked these conditions to autoantibodies, making it difficult to establish causality.

Despite this, the detection of autoantibodies raises important questions about their clinical significance and their potential utility as biomarkers for disease. Future research should focus on disentangling the pathogenic and non-pathogenic roles of these autoantibodies to better understand their implications in post-infection outcomes.

## 7 Conclusion

Autoantibodies have been extensively reported in association with SARS-CoV-2 infections, reinforcing the notion that viral infections, are important environmental factors that can induce *de novo* autoantibody production or amplify existing autoantibody levels. However, there remains a significant gap in our understanding of the precise mechanisms by which these autoantibodies are generated following infection and, more specifically, whether and how they contribute to pathology in the host. It is also plausible that some autoantibodies contribute to regulatory physiological processes during infection or recovery, highlighting their multiple complex roles beyond their known pathogenic effects. Moreover, there is a scarcity of studies exploring therapeutic interventions, such as plasmapheresis or immunosuppressants, specifically targeting these autoantibodies. These gaps in our knowledge represent both challenges and valuable research opportunities. The findings related to SARS-CoV-2 and its association to autoantibodies underscore the complex and intertwined relationship between viral infections and autoimmunity, inviting further exploration into their clinical significance and therapeutic potential.
